# Who wants to be a surgeon? A study of 300 first year medical students

**DOI:** 10.1186/1472-6920-7-2

**Published:** 2007-01-19

**Authors:** Thomas HS Fysh, Geraint Thomas, Harold Ellis

**Affiliations:** 1 Department of Surgery, RCHT Treliske, Treliske, Truro, Cornwall, TR1 3LJ, UK; 2 Department of Surgery, The Royal Free Hospital, Pond St., London NW3 2QG, UK; 3 Department of Anatomy, Hodgkin Building, Guy's Campus, Guy's King's and St Thomas' School of Medicine, St Thomas' Street, London SE1 1UL, UK

## Abstract

**Background:**

While medicine in general is becoming more female-dominated, women are still under-represented in surgery. Opinion is divided as to whether this is due to lifestyle considerations, disinterest or perceived discrimination. It is not clear at what stage these careers decisions are made.

**Methods:**

300 first year medical students at Guy's King's and St Thomas' School of Medicine (GKT) were asked their view on possible career choices at this stage.

**Results:**

While men represented only 38% of the student population, they represented over two-thirds of the students wishing to pursue a career in surgery.

Women still opt for general practice and paediatrics.

**Conclusion:**

Surgery is a disproportionately unpopular career choice of the female first-year medical students of GKT compared to the male students. It appears that the choice is freely made and, at this stage at least, does not represent concerns about compatibility with lifestyle.

## Background

While the medical profession was formerly a male-dominated one, it is now the case that women are actually over represented in medicine [1,2]. The field of surgery, however, is slow to catch up. Although efforts are being employed in the Royal colleges, hospitals and medical schools to reverse this phenomenon, it is still very apparent that, while good results are being achieved recruiting ethnic minorities to surgery, women are still grossly under-represented [1,3-5]. It is conceded that numbers of women entering surgery are rising, but this is at a rate far lower than one would expect [6], with some fields such as orthopaedic and cardiothoracic surgery faring worse than others: A recent study found that while the percentage of female medical students has risen by over 40% since 1970, the proportion of female orthopaedic surgeons has risen by only 8%. Women, it appears, are still opting for general practice and paediatrics.

Some projects have looked into why this trend still exists: The glass ceiling phenomenon is explored in depth [7] along with discrimination [8] and the possibility that women (in general), simply do not want to be surgeons. Results are surprisingly varied. Many convincing studies have found that that lifestyle considerations, training and 'women friendliness' were most important to women (especially when directly questioned) [3,9,10] with some concluding that discrimination and male bias is still rife [3,11]. Others, however, conclude that while these factors are more important to women than to men, women still rate interest and job satisfaction significantly higher than any other motivation [12-17]. A 1996 study of Canadian female surgeons sums up the situation quite well when it suggests that although most female surgeons did not feel actively discriminated against, the perception of discrimination during surgical training was present [11]. Whatever the findings, it is widely accepted that female role models are rare in surgery and that this should be addressed [8,18].

This project aims to discover the career intentions of the first year medical students at Guy's King's and St Thomas' medical school early on in their training. More specifically it aims to determine whether female medical students would like to be surgeons, or whether they prefer other specialties from the outset.

## Methods

300 first year medical students were surveyed in their first two weeks at Guy's, King's and St Thomas' Medical School, London (i.e., before they had had more than a few days exposure to their preclinical teaching). They were surveyed over a two week period after anatomy dissection classes and asked details about their ethnicity, gender and education. They were then asked what they *would like *to specialize in, whether they thought this is what they *would *specialize in and if not, then why not. The questionnaire is shown in the figure. Significance was determined using chi-squared testing.

## Results

All 300 surveys were completed and used (100% response rate).

When compared to the British Medical Association demographics [22], it is seen that the ethnic distribution of GKT Students is significantly different to the UK average for 2003 (X^2 ^> 50, df = 4, P < 0.001). The number of non-white students is higher.

Analysis of the population is shown in table 1. It can be seen that surgery is the single most popular career choice of first year medical students. Medicine and general practice follow, although the proportion of students who are undecided forms almost a quarter of the student body.

**Table 1:** Career Intentions of 300 First-year Medical Students (numbers and percentage choice according to sex, ethnicity and schooling).

***NUMBERS***	**All**	**Men**	**Women**	**Chinese**	**Arab**	**African**	**All Asian**	**Caucasian**	**Not disclosed**	**Post Grad**	**Private**	**State**
**A&E**	1	1	0	0	0	0	0	1	0	0	1	0
**Sports Medicine**	1	1	0	0	0	0	0	1	0	0	0	1
**Anaesthetics**	2	0	2	0	0	0	0	2	0	1	1	1
**Pathology**	3	0	3	0	0	0	0	3	0	1	3	0
**Psychiatry**	8	1	7	0	1	0	3	3	1	2	1	7
**Obs & Gynae**		1	8	0	0	0	2	6	1	0	4	4
**Paediatrics**	20	2	18	3	1	2	2	11	1	3	7	11
**General Practice**	32	7	25	2	1	1	11	17	1	3	11	17
**Medicine**	66	22	44	5	2	6	21	32	2	12	20	35
**Don't Know**	67	22	45	7	2	5	22	27	2	10	24	33
**Surgery**	91	58	33	11	2	6	28	39	4	16	38	33

**Totals**	300	115	185	28	9	20	89	142	12	48	110	142

***PERCENT***	**All**	**Men**	**Women**	**Chinese**	**Arab**	**African**	**All Asian**	**Caucasian**	**Not disclosed**	**Post Grad**	**Private**	**State**

**A&E**	0.3	0.9	0.0	0.0	0.0	0.0	0.0	0.7	0.0	0.0	0.9	0.0
**Sports Medicine**	0.3	0.9	0.0	0.0	0.0	0.0	0.0	0.7	0.0	0.0	0.0	0.7
**Anaesthetics**	0.7	0.0	1.1	0.0	0.0	0.0	0.0	1.4	0.0	2.1	0.9	0.7
**Pathology**	1.0	0.0	1.6	0.0	0.0	0.0	0.0	2.1	0.0	2.1	2.7	0.0
**Psychiatry**	2.7	0.9	3.8	0.0	11.1	0.0	3.4	2.1	8.3	4.2	0.9	4.9
**Obs & Gynae**	3.0	0.9	4.3	0.0	0.0	0.0	2.2	4.2	8.3	0.0	3.6	2.8
**Paediatrics**	6.7	1.7	9.7	10.7	11.1	10.0	2.2	7.7	8.3	6.3	6.4	7.7
**General Practice**	10.7	6.1	13.5	7.1	11.1	5.0	12.4	12.0	8.3	6.3	10.0	12.0
**Medicine**	22.0	19.1	23.8	17.9	22.2	30.0	23.6	22.5	16.7	25.0	18.2	24.6
**Don't Know**	22.3	19.1	24.3	25.0	22.2	25.0	24.7	19.0	16.7	20.8	21.8	23.2
**Surgery**	30.3	50.4	17.8	39.3	22.2	30.0	31.5	27.5	33.3	33.3	34.5	23.2

**Totals**	100.0	100.0	100.0	100.0	100.0	100.0	100.0	100.0	100.0	100.0	100.0	100.0

Data used to derive chi-squared values.

It is seen in table 1 that 18% (33/185) of women on entry into medical school intend to train as surgeons. The null hypothesis that the proportion of women who choose surgery is equal to the proportion of men who wish to do surgery can be tested using a chi squared test with one degree of freedom. Surgery was chosen by 50% of men (33/185). It is found that X^2 ^= 35.7 (P < 0.001). Similarly, a significantly higher proportion of women intend to pursue careers in general practice (X^2 ^= 4.1, df = 1, P < 0.05) and paediatrics (X^2 ^= 7.3, df = 1, P < 0.01), while hospital medicine attracts similar proportions (no significant difference between sexes, X^2 ^= 0.9, df = 1, P > 0.1). Other specialties were difficult to evaluate due to low numbers.

While 22.3% of students did not show a career preference at this stage, 18.2% of students said that they thought they might end up pursuing different careers from those they had initially chosen. By far the most common reason given was that their interests might change (60%), although other areas of concern included competition ability and religion. Family and life-compatibility concerns were mentioned on four occasions, twice for medicine and twice for surgery.

Amongst different ethnicities, there was no significant difference between the observed and expected numbers of students choosing surgery as a career (X^2 ^= 2.0, df = 5, P > 0.1). Numbers were too small to look at each specialty separately, so each ethnic group was divided into 'surgery' and 'not-surgery' for the purposes of this test. Even so, some groups remained small (e.g. for Arab students, n = 9). This affects significance and is discussed in the 'discussion' section. The same was true for education (X^2 ^= 4.5, df = 2, P > 0.1). The chi squared values are derived from the data in table 1.

## Discussion

Our data suggest that while the proportion of women on entry to medical school wishing to pursue a surgical career is around 18% (a high proportion compared to other studies [1,6,19]), this compares to over 50% of male students at the same stage. This is of special concern since men form only 38% of the sample population. Specialties such as general practice and paediatrics continue to attract female medical students.

By allowing students to comment on why they did not think they would pursue a certain line of work, despite an interest in the area, it was possible to conclude that an interest in the line of work was much more important than life-style considerations *at this stage*, confounding the results of other studies [12-14,20] This finding is valuable since it reflects career intentions of students *before *exposure to (and possible bias by) the system; lifestyle considerations *appear *to be less important at this stage and were quoted only four times. It is important to stress that some other studies demonstrate that lifestyle probably becomes an important consideration later on in one's career. This said, a cohort study has shown that while an individual's career preference might change over time, the overall proportions remain similar throughout [21]. It is not our intention to reproduce this finding, but rather to provide an overview of the career intentions of medical students at the earliest possible opportunity in their training. Consequently, no cohort study was carried out. This is discussed further in the 'study limitations' section.

Our study confirms the findings of other groups by showing little difference in the career preferences of graduates compared to non-graduates [19], and shows no significant preference for surgery amongst any ethnic group, although it is conceded that numbers amongst some ethnicities were small and for this finding to be further validated, a larger sample would be desirable. Other study limitations are discussed in the following text.

This survey represents the views of the first year medical students in their first term at medical school; it is the study's express intention to look at the career intentions of this group *before *exposure to the system, and it is accepted that their opinions may change. Although it would certainly be interesting to follow up the sample as a cohort in the years to come as an additional project, it falls beyond the aim of the project at this time.

It should also be noted that the study is set in *one *UK medical school (due to issues of access and practicality) and it is conceded that extra student numbers would lend statistical power to the findings. We were unable to find any other UK studies which looked specifically at the career intentions of first year medical students with which to compare our results. Many of the other studies quoted were completed outside the UK and some time ago. They therefore involved very different student populations and are, consequently, difficult to compare reliably.

It is also alluded to in the 'Results' section that the ethnographic make-up of our student sample is not representative of the UK population as a whole. It is accepted that, while our results are statistically significant, they are not necessarily representative of the UK medical student population as a whole and that every UK medical school would have to be surveyed for our conclusions to be applied nationwide.

It is also noted that the study itself does not detail *why *students chose the careers they did and so it is difficult to comment on this. It does, however, provide comment on why there might be a difference in *intended *as opposed to their own *predicted *future careers. Reasons for *not *choosing a specific career (e.g. lifestyle considerations) were omitted from the questionnaire so as not to lead the responder into a particular answer.

## Conclusion

The primary conclusion to be drawn from our study is that the women surveyed simply do not want to be surgeons: None of the sample group had experienced surgery (and could not, therefore, have been put off the idea) and only two out of nearly 200 women expressed concerns about lifestyle, contrary to convincing evidence gathered from women at *later *stages in their careers. One can only guess as to why this should be but it is perhaps the case that views held prior to entering medical school have lead some women to believe that surgery is an unsuitable option for them.

Although competition and selection is inevitable in a surgical career, being female should be no reason to avoid one. Furthermore, the surgical profession is under increasing pressure to open its doors to a wider selection of medical graduates. The simple argument is, that by increasing the numbers of applicants, one is more likely to appoint the best candidate for the post. If it is the case that gender stereotyping exists from an early age, then it should be discouraged. This has been achieved in medicine as a whole and there is no obvious reason why it could not be done for surgery.

## Competing interests

The author(s) declare that they have no competing interests.

## Authors' contributions

TF is the primary author, conceived of the study, designed it and carried it out. GT carried out the statistical analysis. HE was the department supervisor and acted in an advisory capacity.

**Figure 1:**
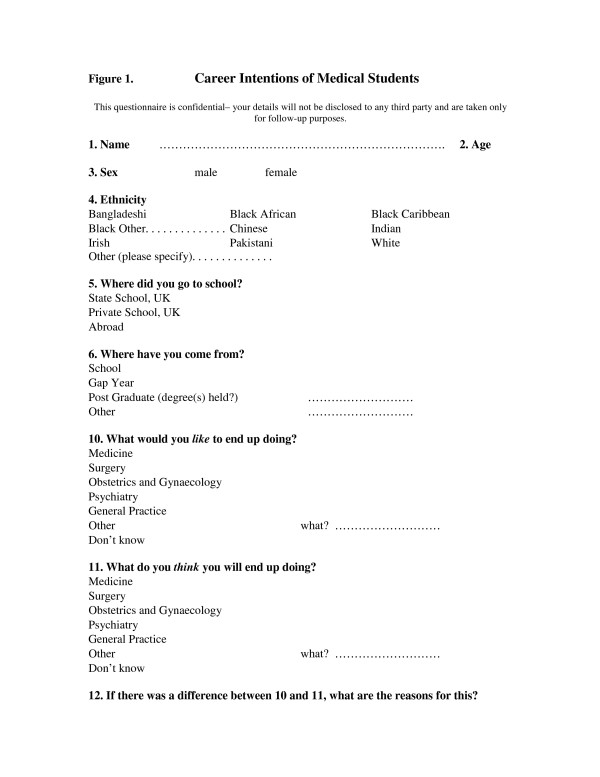
Study Questionnaire.

## Pre-publication history

The pre-publication history for this paper can be accessed here:


